# An Ancient, Light-Dependent Hydrocarbon-Forming Enzyme

**DOI:** 10.1093/plphys/kiab215

**Published:** 2021-07-06

**Authors:** Ananya Mukherjee

**Affiliations:** Plant Science and Innovation, University of Nebraska Lincoln, Lincoln Nebraska, USA

With the rise in energy consumption and desire to cut back on the use of fossil fuels, the demand for renewable energy is increasing every day. Fatty acid-derived hydrocarbons have potential applications as biofuels due to their similarity with petroleum fuels (Liu and Li, 2020). Hydrocarbons are hydrophobic compounds made solely of hydrogen and carbon. In plants, long-chain (C29-35) fatty acid-derived hydrocarbons form the outer waxy layer of the cuticle that prevents dehydration (Sorigué et al., 2016). However, due to their solid-state at ambient temperature, these hydrocarbons cannot be used for our fuel needs. Microbial short-chain hydrocarbons, on the other hand, can be used to create components of diesel, jet fuel, gasoline, cosmetics, and synthons in organic chemistry (Sorigué et al., 2016). Therefore, cyanobacteria and microalgae, which predominantly produce shorter-chain hydrocarbons, are the focus of several research efforts.

In the microalga *Chlorella variabilis* (Cv), *n*-alka(e)nes (straight-chain hydrocarbons with or without double bonds) are produced by decarboxylation of long-chain fatty acids (Sorigué et al., 2017). The (Cv), decarboxylase enzyme requires photons at every catalytic cycle and is thus named fatty acid photo decarboxylase (FAP/CvFAP). Little is known about the role of FAP in microalgae and if it contributes to *n*-alka(e)ne formation. In this issue of *Plant Physiology*, Moulin et al. (2021) investigated the role of FAP in Chlamydomonas (*Chlamydomonas reinhardtii* CrFAP).

To investigate the function of FAP in vivo, Moulin et al. (2021) obtained a *fap* mutant from the Chlamydomonas Library Project. Fatty acid-derived 7-heptadecane, the only known hydrocarbon in Chlamydomonas, was not detected in the *fap* mutant*.* The complemented strains, however, produced wild-type (WT) levels of this hydrocarbon, demonstrating FAP is the only enzyme that produces 7-heptadecane in Chlamydomonas. CrFAP is a soluble protein predicted to localize to the chloroplast, as are FAPs from other red and green algae, and thylakoid purification showed CrFAP bound to this fraction with no stromal protein contamination. 7-heptadecene levels in total fatty acids were significantly enriched in thylakoid fractions as well as when compared to whole cells, and more than 90% of 7-heptadecane localized in thylakoids. Thus, chloroplast-localized CrFAP and its product, 7-heptadecane, closely bind to thylakoid fractions.

Synchronized Chlamydomonas cells showed 7-heptadecene content peaks at the start of the night just before cell division. To determine if FAP plays a role in cell cycle control, growth of the *fap* mutant was compared to that of WT cells. Under standard conditions of growth, cell size, cell division rate, chloroplast structure, and growth rate were similar in the two strains. However, lipidomic analysis showed plastidial lipid content varied between *fap* and WT, in at least three galactolipid species belonging to the digalactosyldiacylglycerol (DGDG) class. Thus, in Chlamydomonas, the absence of FAP influences thylakoid lipid composition, without any obvious effect on growth or cell division.

Under standard conditions (25°C) photosynthetic activity was similar in the *fap* mutant and the WT. Since colder temperatures affect both membrane properties and photosynthetic capacity, both the strains were subjected to a temperature of 15°C and low light. Electron transfer rate (measured at high light intensity) decreased in the *fap* mutant compared to WT at 15°C after a day in low light, whereas maximal photosystem II yield stayed the same. Also, in the WT cells at 15°C, the hydrocarbon content relatively increased compared to WT in 25°C, whereas the rate of cell division and fatty acid production decreased. Because cell division uses hydrocarbons, in colder temperature a decline in the rate of cell division causes the net concentration of hydrocarbons to appear higher. However, it remains to be seen if the net increase in hydrocarbon is an adaptation to colder conditions.

Moulin et al. also conducted a phylogenetic analysis of FAP proteins and found FAP is highly conserved in several algal lineages beyond green algae. FAPs from algae with secondary plastids, red algae, and some macroalgae were especially conserved. Using the TARA Ocean Data (global data on marine plankton from extensive worldwide voyages), they found 198 putative FAPs that can be grouped with CvFAP and have two highly conserved amino acids, Selected FAPs from different algae were heterologously expressed in *Escherichia Coli*. When grown under light conditions (necessary for catalysis by FAPs) these proteins synthesized *n*-alkana(e)s, demonstrating they could potentially be used to manufacture short-chain hydrocarbons for liquid fuel and other biofuel applications.

This study improves upon our current understanding of FAP proteins in microalgae by characterizing the function of CrFAP in vivo. Additionally, 198 new FAPs from green microalgae were identified and several analyzed for FAP activity. These findings have increased our reservoir of FAPs beyond Chlorella for wider application purposes.
